# Echocardiographic evaluation of left ventricular function in patients with mitral regurgitation: a meta-analysis

**DOI:** 10.3389/fcvm.2025.1644591

**Published:** 2025-12-09

**Authors:** Jing Zhang, Qun Qiang

**Affiliations:** Ultrasound Medical Imaging Department, Provincial Hospital of Traditional Chinese Medicine, Lanzhou, Gansu, China

**Keywords:** speckle-tracking echocardiography, mitral regurgitation, GRS, GCS, GLS

## Abstract

**Purpose:**

This study aimed to quantitatively assess left ventricular function in patients with mitral regurgitation (MR) using speckle-tracking echocardiography and further clarify its clinical application.

**Methods:**

PubMed, Embase, Web of Science, and Cochrane Library databases were searched from the date of establishment to November 22, 2024. Two investigators independently screened the literature, extracted data, and evaluated the quality of the included studies. Meta-analysis was performed using Stata18.0 software.

**Results:**

Finally, 23 studies were included, all of which scored ≥7 on the Newcastle–Ottawa scale. Meta-analysis results showed that, the left ventricular end-systolic volume index (*P* < 0.01), left ventricular end-diastolic volume index (*P* < 0.01), and left ventricular mass index (*P* < 0.01) of patients with MR were higher than those of healthy individuals. In contrast, the left ventricular ejection fraction (*P* < 0.01) and global radial strain (GRS) (*P* < 0.01) of patients with MR was lower than that of healthy individuals; the difference was significant. The *E*/*A* of both groups (*P* = 0.22), global circumferential strain (*P* = 0.43), global longitudinal strain (*P* = 0.10), left ventricular relative wall thickness (*P* = 0.20), and some indexes with significant heterogeneity were analyzed by subgroup according to the degree of MR. The combined results of most subgroup analysis were consistent with the overall results and the heterogeneity was reduced.

**Conclusion:**

There is significant difference in the GRS between patients with MR and healthy individuals, which can provide reference for evaluating left ventricular function in patients with MR.

## 1 Introduction

Mitral regurgitation (MR) refers to a series of cardiac pathophysiological changes and clinical symptoms caused by congenital abnormalities or acquired diseases that prevent the mitral valve from completely closing during left ventricular contraction, resulting in blood regurgitation from the left ventricle back to the left atrium. It is one of the most common heart valve diseases (HVDs). Severe MR, if not corrected in time, will cause progressive cardiac dysfunction. In addition to the requirements of long follow-up and large investigation and treatment costs in the later stages of HVD, owing to the close relationship between HVD and age, the prevalence of MR will also increase rapidly as the population ages, bringing new challenges to the global health care system (1). Speckle-tracking echocardiography (STE) uses high-frame two-dimensional gray-scale images to identify small, stable myocardial footprints or spots generated by ultrasound-myocardial tissue interaction and to obtain Doppler information about global and segmental myocardial deformation by tracking the distance or spatial displacement between spots during cardiac motion in real time (2). STE relies on measuring two-dimensional intra-tissue velocity to distinguish between normal myocardial segment motion and myocardial motion when adjacent myocardial segments are restricted or when motion of the entire heart is dysfunctional. Compared with conventional tissue Doppler imaging, STE is more consistent with Lagrangian strain theory and compared with its initial length (2). STE measurement of left atrial strain is a new detection method that has been developed in recent years. Two-dimensional STE analysis of the left atrium can obtain measurement results of atrial strain and volume, which can improve the accuracy and repeatability of left ventricular diastolic function evaluation and shorten the reporting time, which has important clinical value (3–5). Studies have shown that STE may predict cardiac function earlier than ejection fraction (6). This study aimed to evaluate left ventricular function in patients with MR using echocardiography and speckle-tracking technique and to provide a new reference for clinical diagnosis.

## 2 Methods

### 2.1 Literature search

PubMed, Embase, Web of Science, and Cochrane library databases were searched for controlled studies on left ventricular function in patients with MR evaluated by STE from the date of establishment to November 22, 2024.

### 2.2 Inclusion and discharge standards

The inclusion criteria were as follows: (1) study type: control study of patients with MR; (2) study object: clear MR diagnostic criteria; (3) measurement method and evaluation index: Use of speckle-tracking technique to assess left ventricular function and measure global longitudinal strain (GLS), global circumferential strain (GCS), global radial strain (GRS), left ventricular ejection fraction (LVEF), left ventricular end-diastolic volume index (LVEDVi), left ventricular end-systolic volume index (LVESVi), *E*/*A*, relative wall thickness (RWT), and left ventricular mass index (LVMI).

### 2.3 Exclusion criteria

We excluded (1) reviews, case reports, and abstracts from academic conferences; (2) repeated published or included studies, retaining articles with large sample size and comprehensive information; (3) studies without a healthy control group; (4) study participants suffering from valve diseases other than MR, such as tricuspid regurgitation; (5) studies for which full text or overall valid data could not be obtained.

### 2.4 Literature screening and data extraction

The basic data extracted included the first author's name, country, publication year, number of MR cases included, number of control groups, basic characteristics of the MR and control group populations (sex, age, smoking history, body mass index, history of hypertension and diabetes, etc.), conventional echocardiography, and speckle-tracking technology measurement parameters (LVEF, GLS, GRS, GCS, LVEDVi, LVESVi, *E*/*A*, RWT, and LVMI).

### 2.5 Quality evaluation

The Newcastle–Ottawa scale recommended by the American Institute for Health Care Research and Quality was used to evaluate the quality of the literature (Table 1).

**Table 1:** Bias risk evaluation of the included studies.

Study (first author and year of publication)	Selection	Comparability	Outcome	Total score
Bakkestrøm et al. (6)	★★★★	★★	★★	7★
Cameli et al. (7)	★★★★	★★	★★★	9★
Borg et al. (8)	★★★★	★★	★★★	9★
Casas et al. (9)	★★★★	★	★★★	8★
Dirsiene et al. (10)	★★★★	★★	★★★	9★
Ermakov et al. (11)	★★★★	★★	★★★	9★
Gelsomino et al. (12)	★★★	★★	★★★	8★
Florescu et al. (13)	★★★★	★★	★★★	9★
Huttin et al. (14)	★★★★	★★	★★★	9★
Kawase et al. (15)	★★★★	★★	★★★	9★
Luca et al. (16)	★★★	★★	★★	7★
Kılıcgedik et al. (17)	★★★★	★★	★★★	9★
Lancellotti et al. (18)	★★★★	★★	★★★	9★
Mihaila et al. (19)	★★★★	★★	★★★	9★
Rajesh et al. (20)	★★★	★★	★★★	8★
Sonaglioni et al. (21)	★★★	★★	★★	7★
Sugimoto et al. (22)	★★★★	★★	★★★	9★
Valuckiene et al. (23)	★★★	★★	★★★	8★
Tang et al. (24)	★★★★	★★	★★★	9★
Witkowski et al. (25)	★★★★	★★	★★★	9★
Zvirblyte et al. (26)	★★★	★★	★★	7★
Debonnaire et al. (27)	★★★★	★★	★★★	9★
Cameli et al. (28)	★★★	★★	★★★	8★

### 2.6 Statistical analysis

Meta-analysis was performed using Stata18.0 software. A heterogeneity test (*Q* test) was performed to evaluate the heterogeneity among the included studies. *I*^2^ > 50% or *P* < 0.1 was considered as indicative of significant heterogeneity among the included studies. Meta-analysis was performed using a random-effects model; otherwise, a fixed-effects model was used. A sensitivity analysis was performed to determine the stability of the study results by removing significant changes in observations based on individual articles. Statistical indicators used included weighted mean difference (WMD) and 95% confidence interval (CI).

## 3 Results

**Figure 1:**
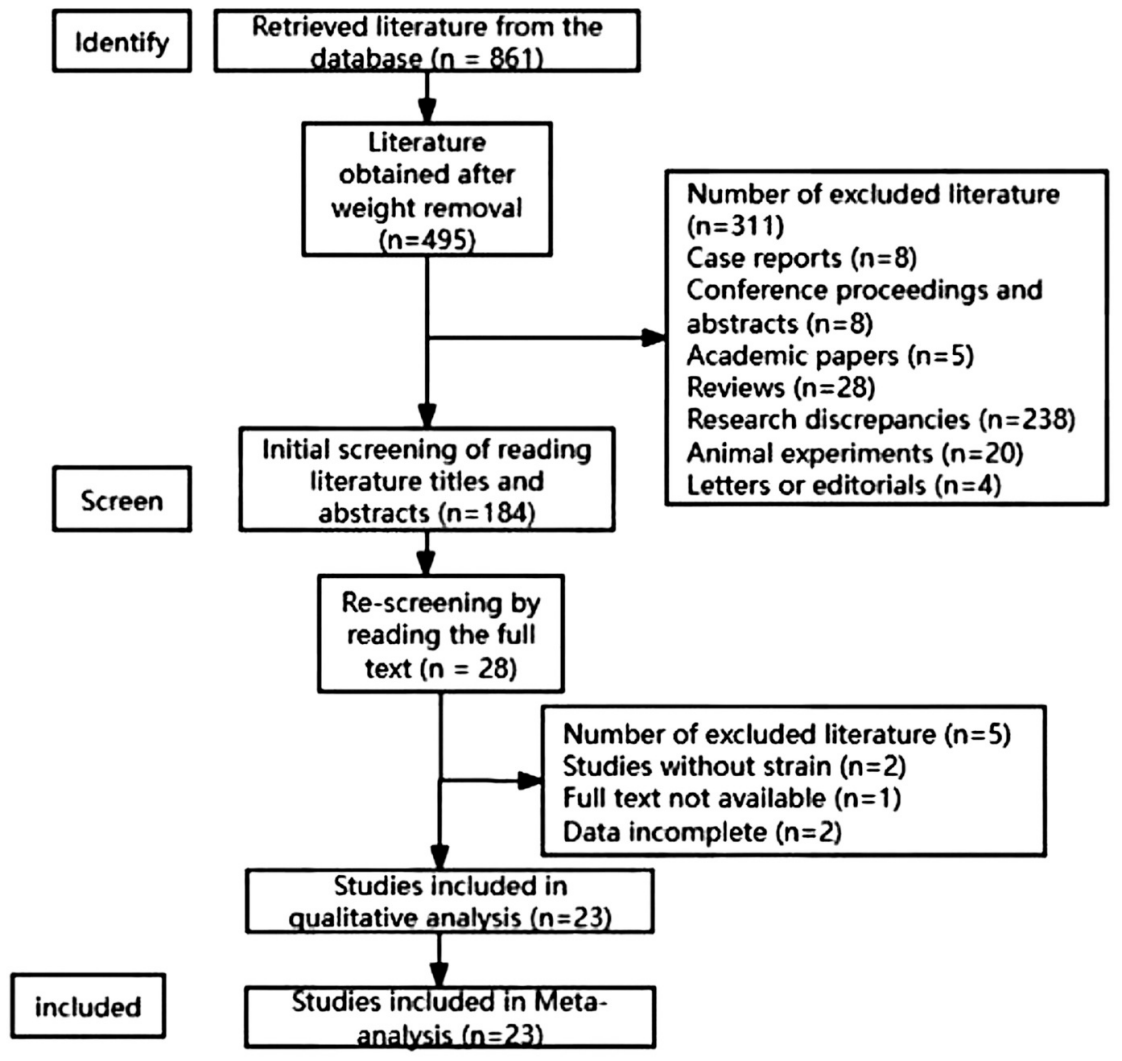
Flow chart of literature retrieval.

1. A total of 843 articles were obtained from the preliminary search and 23 that met the inclusion criteria were finally included (Figure 1). And the baseline date of all included studies shown in Table 2.
2. Meta-analysis results.

**Table 2:** Basic characteristics of the included studies (case/control groups).

Study (first author and year of publication)	Sample size	Sex (male)	Age (years)	Body surface area (m^2^)	BMI	Smoker	Atrial fibrillation	Hypertension/blood pressure	Diabetes mellitus	Dyslipidemia
Bakkestrøm et al. (6)	66	34/12	64 ± 9/56 ± 17	1.98 ± 0.18/1.89 ± 0.19	NR	8/0	NR	17/0	6/4	NR
Cameli et al. (7)	200	68/28	55.73 ± 12.34/55.6 ± 7.5	1.78 ± 0.19/1.81 ± 0.24	24.9 ± 4.1/24.7 ± 5.1	24/12	NR	125.9 ± 12.8/78.8 ± 8.5/122.5 ± 13.5/77.6 ± 9.4	NR	NR
Borg et al. (8)	68	NR	64.6/60.1	1.83/1.77	NR	NR	NR	145/82 146/84	NR	NR
Casas-Rojo et al. (9)	65	NR	NR	NR	NR	NR	NR	NR	NR	NR
Ermakov et al. (10)	98	29/18	52/55	1.87/1.80	NR	NR	12/13	13/5	0/0	NR
Gelsomino et al. (11)	81	40/14	66.8 ± 6.6/66.0 ± 4.4	1.87 ± 1.7/1.85 ± 1.6	25.2 ± 1.6/23.3 ± 1.2	NR	NR	29/	16	NR
Florescu et al. (12)	38	NR	59 ± 13/61 ± 7	NR	NR	NR	NR	NR	NR	NR
Huttin et al. (13)	120	70/16	58.3 ± 14.7/58.8 ± 9.2	24.1 ± 3.7	NR	NR	NR	NR	NR	NR
Kawase et al. (14)	108	34/19	67.6 ± 9.0/67 ± 11	1.64 ± 0.19/1.66 ± 0.18	NR	13/4	24/	NR	23/0	18/
Luca et al. (15)	115	56	66.4 ± 6.3	NR	NR	NR	NR	39/	26	NR
Kılıcgedik et al. (16)	94	49/20	54.3 ± 15/52.7 ± 9.4	NR	NR	NR	13/0	0/0	9/3	NR
Kim et al. (17)	93	46/21	54.2 ± 13.9/53.7 ± 8.3	NR	NR	NR	30	NR	NR	NR
Lancellotti et al. (18)	94	37/10	60.95 ± 14.4/58.4 ± 11	NR	NR	NR	NR	NR	NR	NR
Mihaila et al. (19)	104	31/31	57 ± 15/56 ± 13	1.75 ± 0.2/1.82 ± 0.2	NR	NR	NR	132 + 20/78 + 10,127 + 17/74 + 8	NR	NR
Rajesh et al. (20)	100	NR	NR	NR	NR	NR	NR	NR	NR	NR
Sonaglioni et al. (21)	120	32/30	50.1 ± 8.6/50.6 ± 11.3	1.81 ± 0.16/1.83 ± 0.15	NR	10/8	NR	10/8	6/4	15/12
Sugimoto et al. (22)	250	56/50	66.4 ± 12.8/59.5 ± 13.3	NR	25.9 ± 4.5/25.8 ± 3.8	35/26	NR	65/63	21/17	49/39
Valuckiene et al. (23)	104	46/21	61.13 ± 11.64/57.3 ± 6.1	NR	28.2 ± 4.2/26.6 ± 3.2	44/10	NR	41/0	NR	52/13
Tang et al. (24)	86	28/8	70.3 ± 8.4/69 ± 8	1.7 ± 0.2/1.7 ± 0.2	NR	NR	54/NA	NR	NR	NR
Witkowski et al. (25)	162	76/24	60/60	1.95/1.89	NR	NR	15	32	5	NR
Zvirblyte et al. (26)	80	13/10	61.88 ± 12.88/57.4 ± 10.89	1.81 ± 0.19/1.88 ± 0.17	NR	2/1	14/1	31/18	2/2	NR
Debonnaire et al. (27)	191	77/47	63 ± 13/63 ± 12	1.9 ± 0.2/1.9 ± 0.21	NR	21/25	26/0	11/31	4/9	11/20
Cameli et al. (28)	168	58/27	68.2 ± 10.4/64.4 ± 15.5	1.71 ± 0.59/1.77 ± 0.2	24.96 ± 4.1/24.2 ± 2.2	NR	NR	121.5 ± 7.7/77.5 ± 7.2	NR	NR

BMI, body mass index; NR, not reported.

### 3.1 LVEF

**Figure 2:**
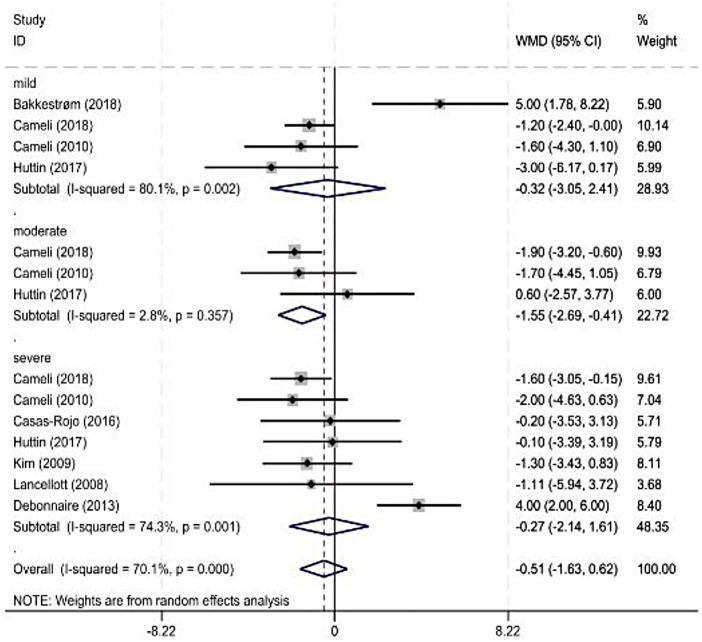
Forest plot of left ventricular ejection fraction.

A total of 21 studies (7–20, 22–25, 27–29) provided the LVEF results. As the heterogeneity among these studies was significant, meta-analysis was performed using a random-effects model. The results showed that the LVEF of the healthy individuals was significantly higher than that of patients with MR (*P* < 0.05, *I*^2^ = 95%; Figure 2).

### 3.2 GLS

Nine studies (7, 8, 10, 11, 13, 17, 21, 22, 26) provided the GLS results. As the heterogeneity among these studies was significant, meta-analysis was performed using a random-effects model. The results indicated no significant differences in the GLS between healthy individuals and patients with MR (*P* > 0.05).

### 3.3 GRS

Three studies (10, 13, 21) provided the GRS results. As the heterogeneity among these studies was not significant, a fixed-effects model was used for meta-analysis. The results showed that the GRS of healthy individuals was significantly higher than that of patients with MR (*P* < 0.05).

### 3.4 GCS

Four studies (10, 21, 22, 26) provided the GCS results. As the heterogeneity among these studies was significant, a random-effects model was used for meta-analysis. The results showed no significant differences in the GCS between healthy individuals and patients with MR (*P* = 0.43, *I*^2^ = 93%).

### 3.5 LVEDVi

Nine studies (9, 11, 12, 22–26, 28) provided the LVEDVi results. Because of the significant heterogeneity among these studies, meta-analysis was performed using a random-effects model. The results showed that the LVEDVi of patients with MR was significantly higher than that of healthy individuals (*P* < 0.01, *I*^2^ = 98%).

### 3.6 LVESVi

Eight studies (7, 11, 12, 16, 24–26, 28) provided the LVESVi results. Meta-analysis was performed using a random-effects model owing to significant heterogeneity among these studies. The results showed that the LVESVi of patients with MR was significantly higher than that of healthy individuals (*P* < 0.01; *I*^2^ = 99%).

### 3.7 LVMI

Eight studies (7–9, 11, 22–24, 29) provided the LVMI results. Because of the significant heterogeneity among these studies, meta-analysis was performed using a random-effects model. The results showed that the LVMI of the healthy individuals was significantly lower than that of patients with MR (*P* < 0.01, *I*^2^ = 90%).

## 4 A subgroup analysis

**Figure 3:**
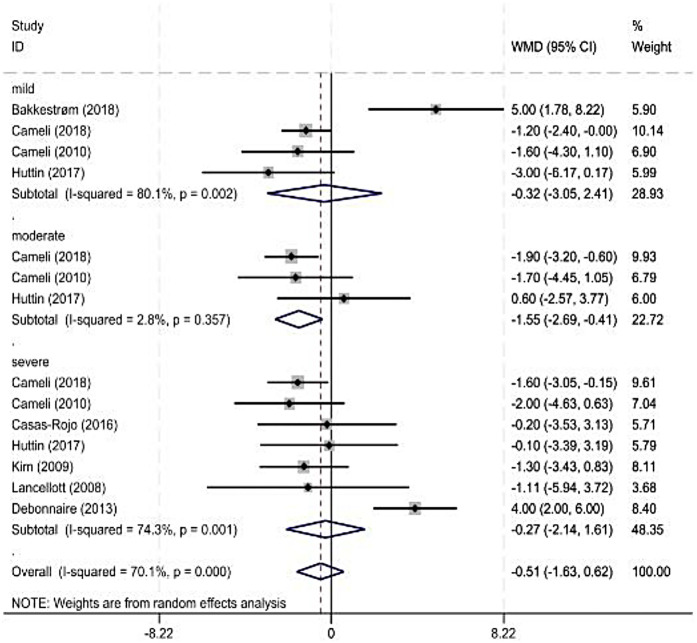
Forest plot of left ventricular ejection fraction subgroup.

As some observation indicators were significantly heterogeneous among the included studies, a subgroup analysis was performed for each observation indicator according to the severity classification of MR (American Society of Echocardiography standard). The main observation indicator was the LVEF, and its results are shown in Figure 3 and Table 3. The combined results for other indicators with reduced heterogeneity are also shown in Table 3.

**Table 3:** Results of meta-analysis of outcome indicators.

Outcome indicator	Number of included studies	Heterogeneity test results		Effect model	Meta-analysis results	
		*P*	*I*^2^ (%)		*OR/WMD* (*95% CI*)	*P*
*E*/*A*	5	<0.01	96	Random	0.19 (−0.11, 0.49)	0.22
Mild	2	0.08	68	Random	−0.31 (−0.52, −0.11)	<0.01
Moderate	2	0.79	0	Random	−0.30 (−0.41, −0.19)	<0.01
Severe	2	0.38	0	Random	−0.24 (−0.39, −0.09)	<0.01
GCS	4	<0.01	94	Random	2.88 (−1.29, 7.06)	0.43
GLS	9	<0.01	94	Random	1.43 (−0.26, 3.12)	0.10
GRS	3	0.98	0	Fixed	−8.55 (−9.11, −7.00)	<0.01
LVEF	21	0.00	95	Random	−3.82 (−6.20, −1.45)	<0.01
Mild	4	<0.01	80	Random	−0.32 (−3.05, 2.41)	0.82
Moderate	3	0.36	3	Random	−1.55 (−2.69, −0.41)	0.01
Severe	8	<0.01	88	Random	−1.66 (−4.28, 0.96)	0.78
LVESVi	8	<0.01	99	Random	12.45 (4.05, 20.85)	<0.01
LVEDVi	9	<0.01	98	Random	17.72 (7.53, 27.90)	<0.01
RWT	5	<0.01	96	Random	0.04 (−0.02, 0.10)	0.20
Mild	3	<0.01	99	Random	0.26 (−0.02, 0.54)	0.06
Moderate	3	<0.01	100	Random	−0.11 (−0.36, 0.14)	0.39
Severe	3	<0.01	97	Random	0.04 (−0.08, 0.17)	0.49
LVMI	8	<0.01	90	Random	31.08 (22.92, 41.25)	<0.01

OR, odds ratio; WMD, weighted mean difference; CI, confidence interval; GCS, global circumferential strain; GLS, global longitudinal strain; GRS, global radial strain; LVEF, left ventricular ejection fraction; LVESVi, left ventricular end-systolic volume index; LVEDVi, left ventricular end-diastolic volume index; RWT, relative wall thickness, LVMI, left ventricular mass index.

### 4.1 LVEF

Four (7, 14, 29) and eight studies were included in the mild and severe subgroups, respectively, in the LVEF subgroup analysis according to the degree of MR. A random-effects model was used for the analysis. The results showed no significant difference in the LVEF between healthy individuals and patients with MR. Three studies were included in the moderate-MR subgroup. Heterogeneity was significant; therefore, a random-effects model was used. The results showed a significant difference in the LVEF between healthy individuals and patients with MR.

### 4.2 E/A

Subgroup analysis of *E*/*A* according to the degree of MR was performed and two studies (8, 29) were included. The heterogeneity of *E*/*A* across different severities of MR was significant; therefore, a random-effects model was used for the analysis. The results of the subgroup analysis showed that the *E*/*A* of patients with mild, moderate, and severe MR were significantly lower than those of healthy individuals (*P* < 0.01), and the heterogeneity was significantly reduced.

### 4.3 RWT

Three studies (7, 8, 29) were included in the subgroup analysis of left ventricular RWT. The results of the subgroup analysis showed no significant difference in left ventricular RWT between healthy individuals and patients with different degrees of MR, and there was no significant change in heterogeneity.

## 5 Sensitivity analysis

Sensitivity analysis of each observation index with significant heterogeneity was carried out by eliminating one study at a time. The results showed that The results did not change significantly, suggesting that they were stable.

## 6 Publication bias

Funnel plots were constructed using Stata18.0 software, and publication bias was identified using the Egger's test. The funnel plots of all observation indicators were not significantly asymmetric, and the publication bias results of the observation indicators obtained using the Egger's test were as follows: GLS, LVEF, GRS, GCS, LVMI, RWT, LVEDVi, *E*/*A*. The above data showed that there was no significant publication bias in the above indexes. However, LVESVi (*P* = 0.03) showed a publication bias. Further analysis using the clipping method showed that the random-effects result after adding one dummy data point [Hedges = 1.12, 95% CI (0.29,1.97)] was not reversed with the random-effect outcome of LVESVi before adding the dummy data point [Hedges = 1.29, 95% CI (0.48,2.10)]; therefore, although there was publication bias for this index, the outcome was still robust.

## 7 Discussion

MR is a common HVD. Given the rapid aging of the global population, HVD is expected to become the next “heart epidemic.” Accurately assessing the cardiac function of patients with MR is important to assist in clinical diagnosis and early intervention. Therefore, improving the prognosis of patients with MR is crucial. This study analyzed the cardiac function of patients with MR using STE. Nakagawa et al. (30) proposed that left ventricular function was impaired and end-diastolic volume index was increased in patients with MR, which was consistent with the results of our study that LVEDVi [WMD = 21.29, 95% CI (0.49, 32.09), *P* < 0.01, *I*^2^ = 98%] of patients with MR was higher than that of healthy individuals. Other studies have demonstrated (31) that a strong linear relationship exists between valvular regurgitation volume and LVEDVi and that left ventricular volume better reflects ventricular remodeling in patients with MR. LVESVi is associated with mortality and symptoms in patients with aortic regurgitation (32). We suspect that LVESVi can also be used to assess cardiac function in patients with MR. Our meta-analysis results showed that LVESVi [WMD = 12.45, 95% CI (−4.05, 20.85), *P* < 0.01, *I*^2^ = 99%] of patients with MR was significantly higher than that of healthy individuals. A recent study (33) reported that the LVESVi was an independent predictor of postoperative left ventricular dysfunction in patients with chronic degenerative MR. This study further illustrates the importance of LVESVi for the assessment of left ventricular function in patients with MR. In addition, our results showed that the LVMI [WMD = 32.08, 95% CI (22.92, 41.25), *P* < 0.01, *I*^2^ = 90%] and LVEF [WMD = −3.82, 95% CI (−6.20, −1.45), *P* < 0.01, *I*^2^ = 95%] of patients with MR was significantly lower, whereas their GRS [WMD = −8.55, 95% CI (−9.11, −7.00), *P* < 0.01, *I*^2^ = 0%] was significantly lower, compared with healthy individuals. These results indicate reduced cardiac function in patients with MR, which is consistent with the results from the study by Shechter et al., who argued that LVEF decline in patients with MR was driven primarily by reduced total stroke volume and diffuse myocardial contractility (34). Previous studies have shown that the GLS (35) can directly reflect myocardial motion and better assess myocardial function. The GLS is lower in symptomatic patients undergoing transcatheter aortic valve replacement. The GCS is positively correlated with ejection fraction, whereas atrioventricular valve regurgitation is negatively correlated with it (6). The RWT is considered (36) an index for evaluating ventricular hypertrophy; however, it cannot distinguish patients with aortic regurgitation from healthy individuals. In our study, we found no significant difference in the *E*/*A*, GCS, GLS or RWT between patients with MR and healthy individuals, which is inconsistent with the results of previous studies. This may be because of the small number and small sample size of the included studies, and the results of this study were affected by baseline data, such as sex and age. Subgroup analysis of some indicators with significant heterogeneity according to the degree of MR showed that the combined results of most subgroup analyses of the observed indicators were consistent with the overall results, and the heterogeneity was reduced. Cardiac strain is affected by sex and age, with one study in 2011 showing a significant decrease in strain values with age (37) and another showing lower strain values in healthy men than in women (38). Sex-stratified analysis was not performed in this study, and the influence of sex on the results cannot be excluded. Further studies stratifying data based on confounding factors, such as sex and age, are needed. Some studies have shown differences in the cardiac strain measured using different ultrasound techniques and software, whereas others have shown insignificant differences based on such factors. However, further studies are needed to confirm this view.

MR is often divided into ischemic (such as mitral regurgitation caused by coronary artery disease) and non-ischemic (all other causes) according to the cause. According to the mechanism, it can be divided into functional (normal mitral valve structure, valve deformation caused by ventricular remodeling) and organic (valve itself disease) (39), this study included The original studies entered did not group different mechanisms or causes of mitral regurgitation, but only made simple frequency statistics, and most studies did not involve different mechanisms or causes. There was insufficient data to support subgroup analysis. Therefore, more studies are needed to analyze the impact of different causes or mechanisms of mitral regurgitation on cardiac function. In addition, according to different causes and mechanisms, the choice of intervention measures for patients with mitral regurgitation is different. The use of drugs and surgical repair will affect the prognosis of mitral regurgitation and improve atrioventricular remodeling, thereby further improving the strain index and atrioventricular function (40). Due to the pathological changes in the valve itself, the use of drugs in organic mitral regurgitation is relatively functional. The effect in patients with cusp regurgitation, especially ischemic mitral regurgitation, was not significant, which affected the results of our study. Some of the people included in this study used drugs to improve cardiac function, but the original study did not explain the use of drugs in detail, which made it impossible to conduct a comparative analysis between the drug group and the non-drug group, which also led to the limitations of the study. In addition, some indicators, such as the GLS and GCS, were rarely included in the statistical analysis of this study, and the sample size was small; therefore, the study results have certain limitations. Although this study has some limitations, the results are consistent with observations from clinical practice and are reliable.

In addition, transesophageal echocardiography (TEE) can also evaluate mitral valve structure and function. As an important tool for mitral valve repair surgery, TEE can determine the mechanism and severity of mitral regurgitation, as well as assess the severity of residual regurgitation and the diagnosis of other complications after surgery. For minimally invasive mitral valve surgery, TEE can also evaluate the position of extracorporeal circulation cannulation (41–43). Further studies could incorporate TEE to evaluate cardiac function in patients with mitral regurgitation.

To address the above limitations, larger sample sizes and more studies are needed to explore the reliability of myocardial strain in evaluating cardiac function in patients with mitral regurgitation. It is expected to detect cardiac function abnormalities earlier than the reduction of LVEF, thereby guiding diagnosis and treatment.

## 8 Conclusion

Conventional echocardiographic parameters, such as the LVEF, LVEDVi, LVESVi, and LVMI, can assess ventricular function in patients with MR, and the myocardial strain parameter, GRS, obtained using speckle-tracking technology can also assess left ventricular function in patients with MR, providing more comprehensive evidence for monitoring cardiac function.

## Data Availability

The original contributions presented in the study are included in the article/Supplementary Material, further inquiries can be directed to the corresponding author.
