# Supplementary figures and images for: Echocardiographic evaluation of left ventricular function in patients with mitral regurgitation: a meta-analysis
Source: Front Cardiovasc Med. 2025 Dec 9;12:1644591. doi: 10.3389/fcvm.2025.1644591 (PMC12723008; doi:10.3389/fcvm.2025.1644591)

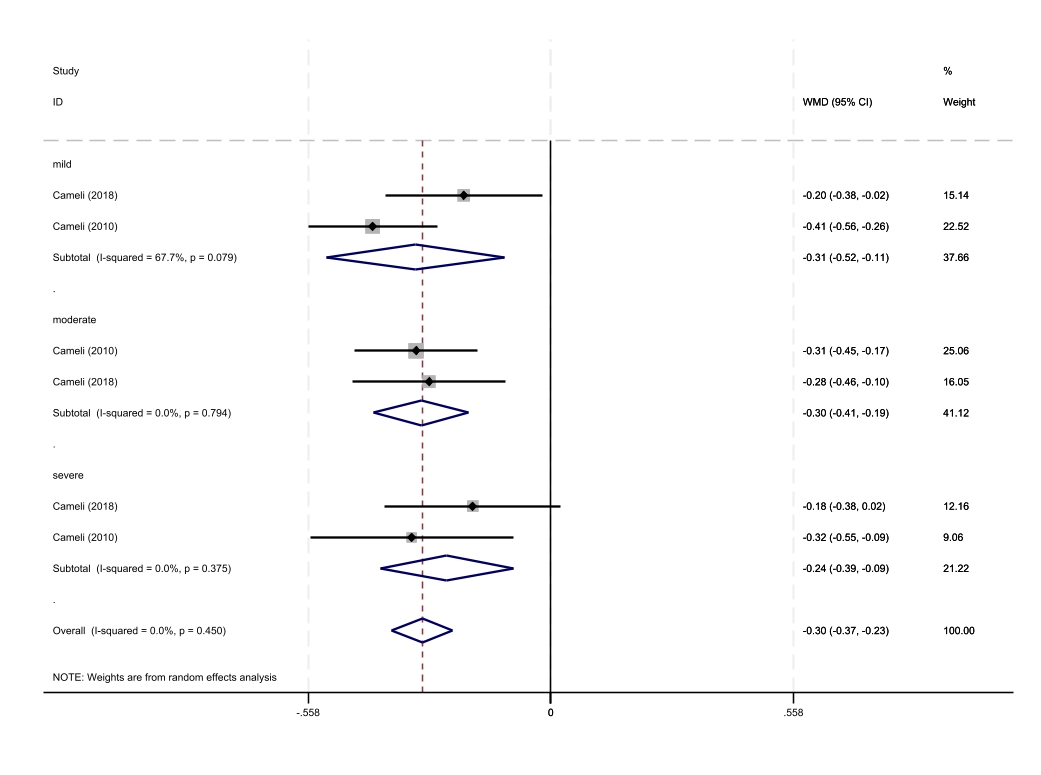

Supplement: Supplementary file 1 [file Image1.jpeg]

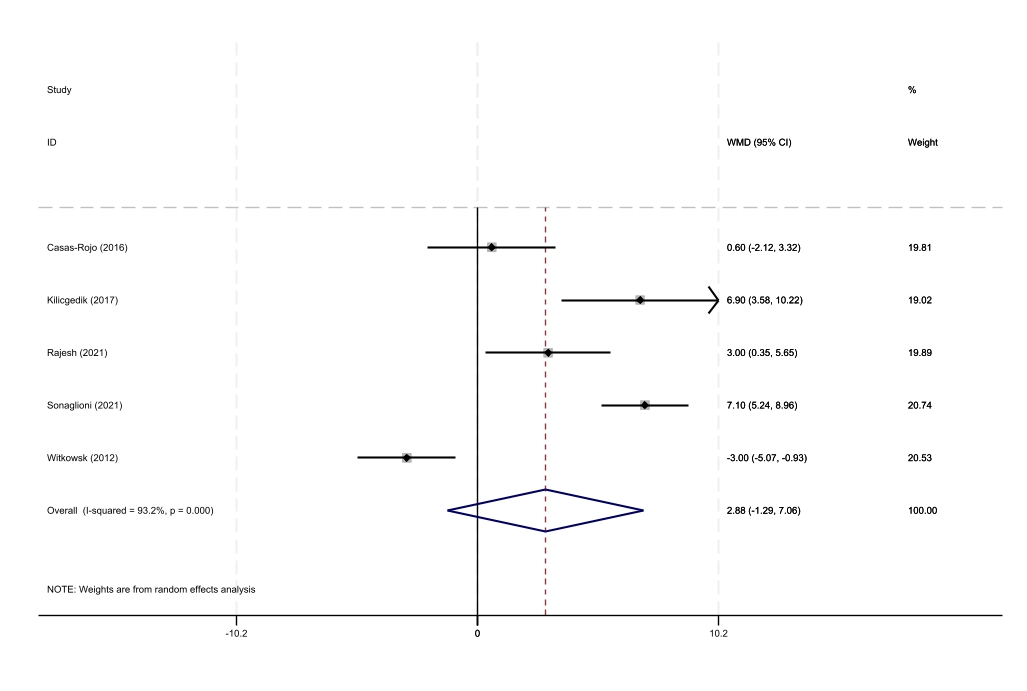

Supplement: Supplementary file 2 [file Image2.jpeg]

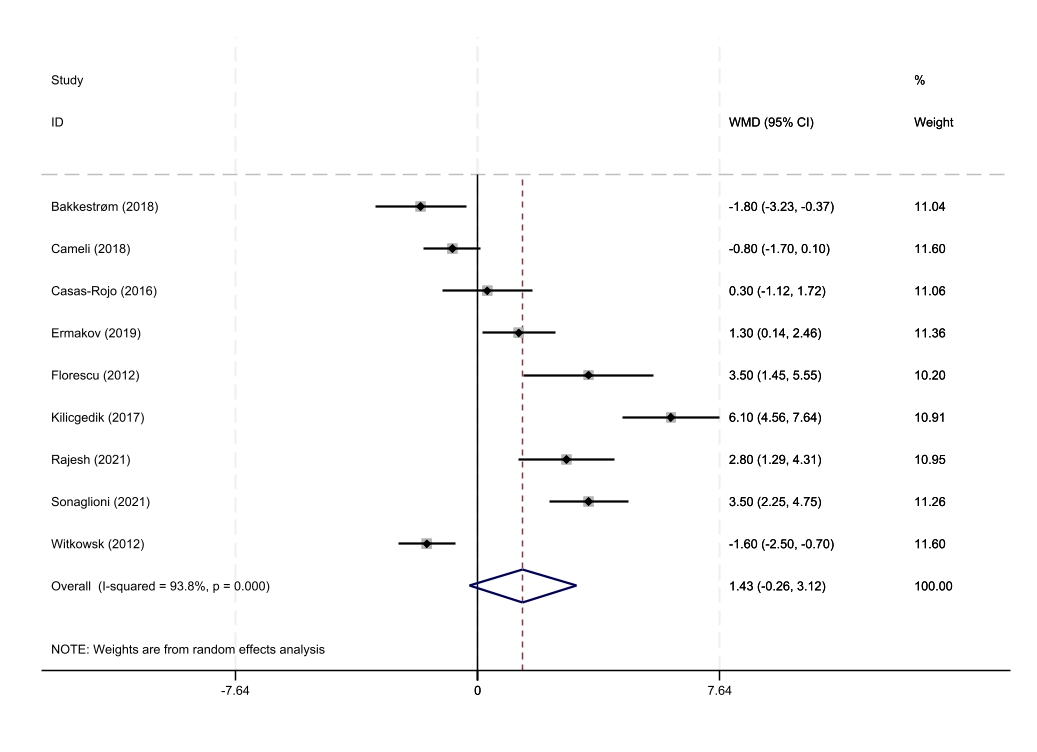

Supplement: Supplementary file 3 [file Image3.jpeg]

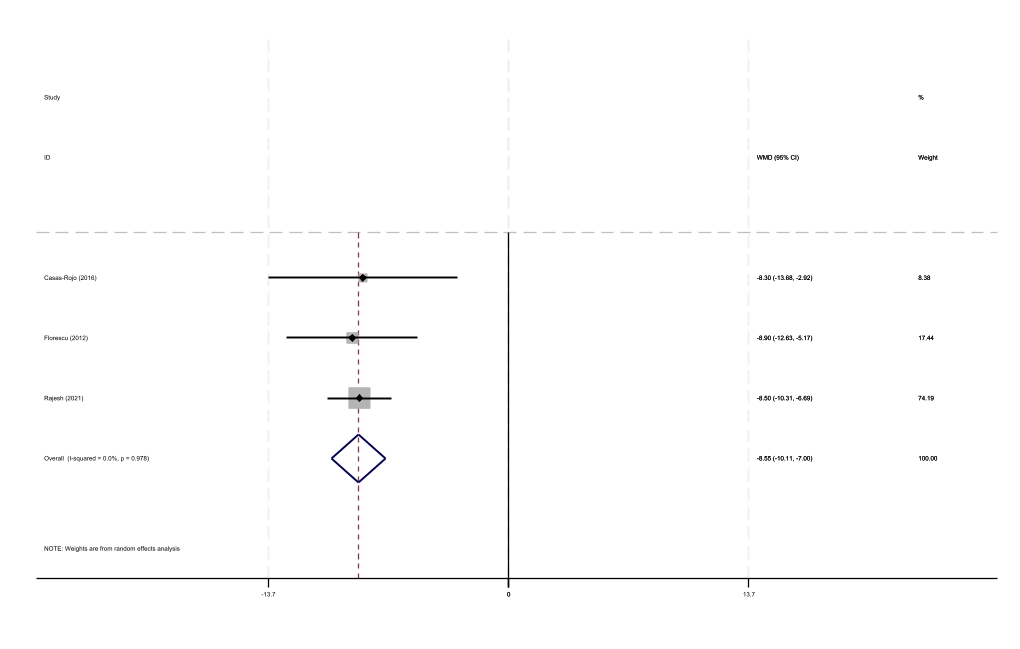

Supplement: Supplementary file 4 [file Image4.jpeg]

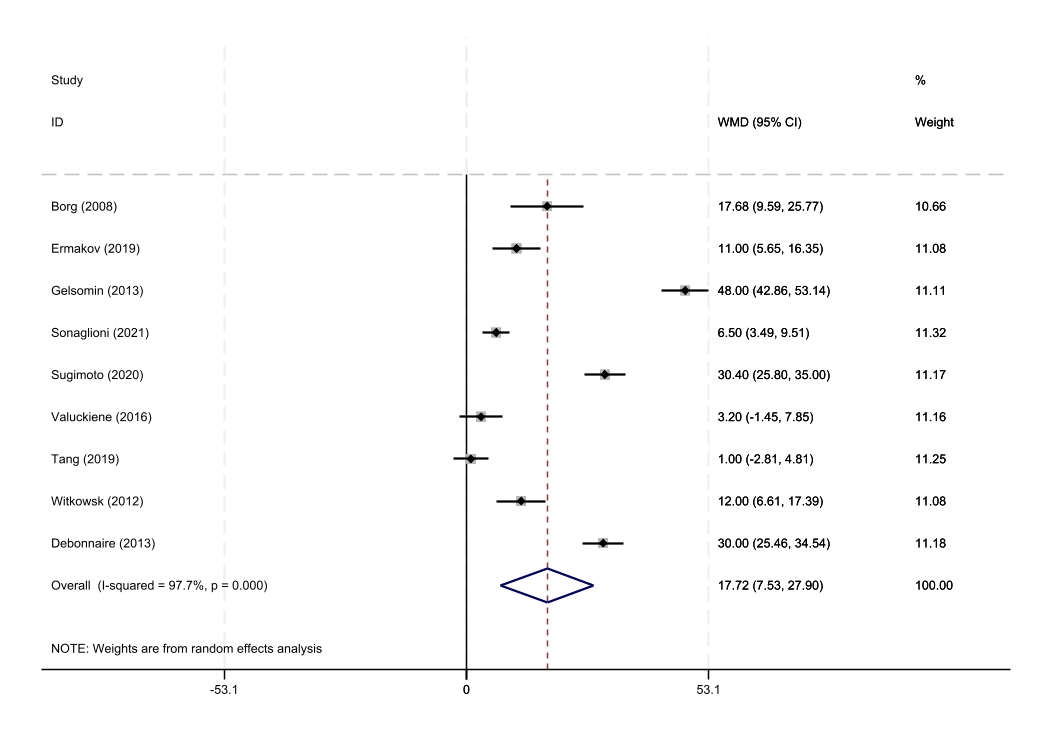

Supplement: Supplementary file 5 [file Image5.jpeg]

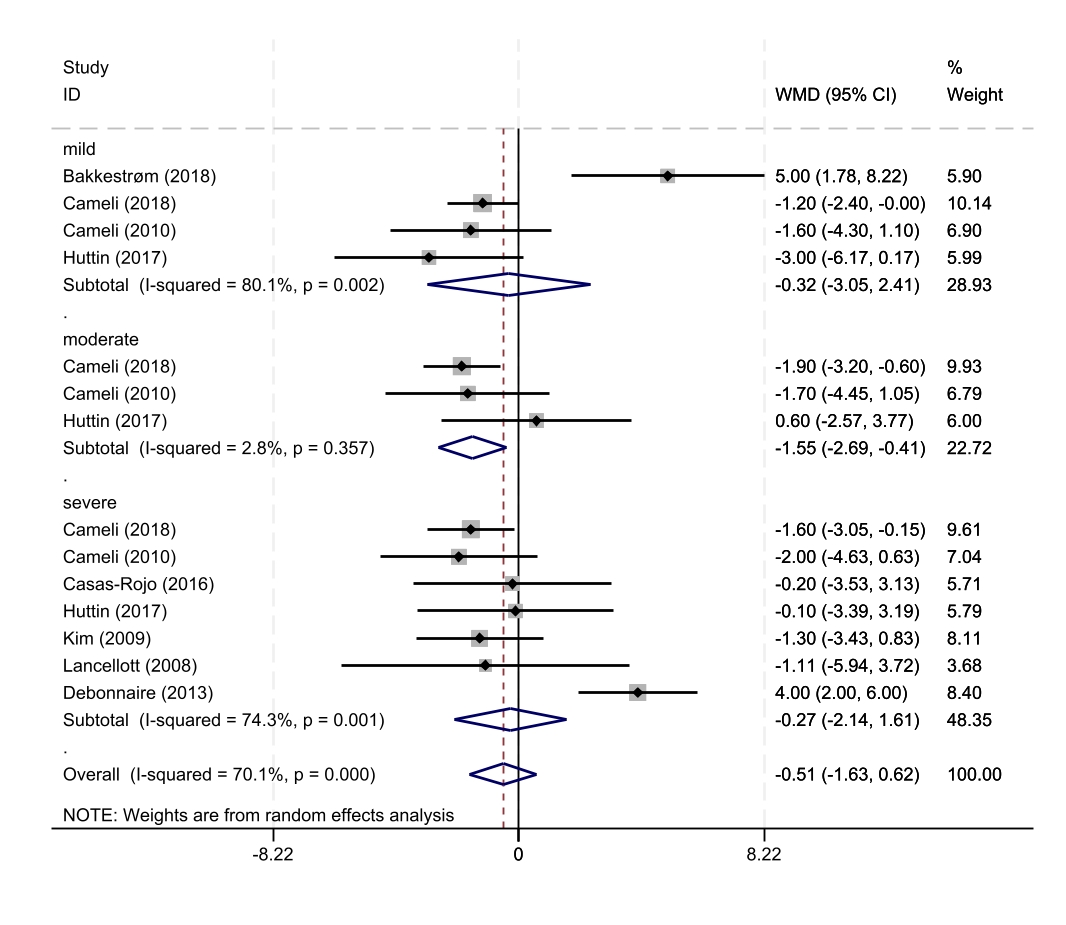

Supplement: Supplementary file 6 [file Image6.jpeg]

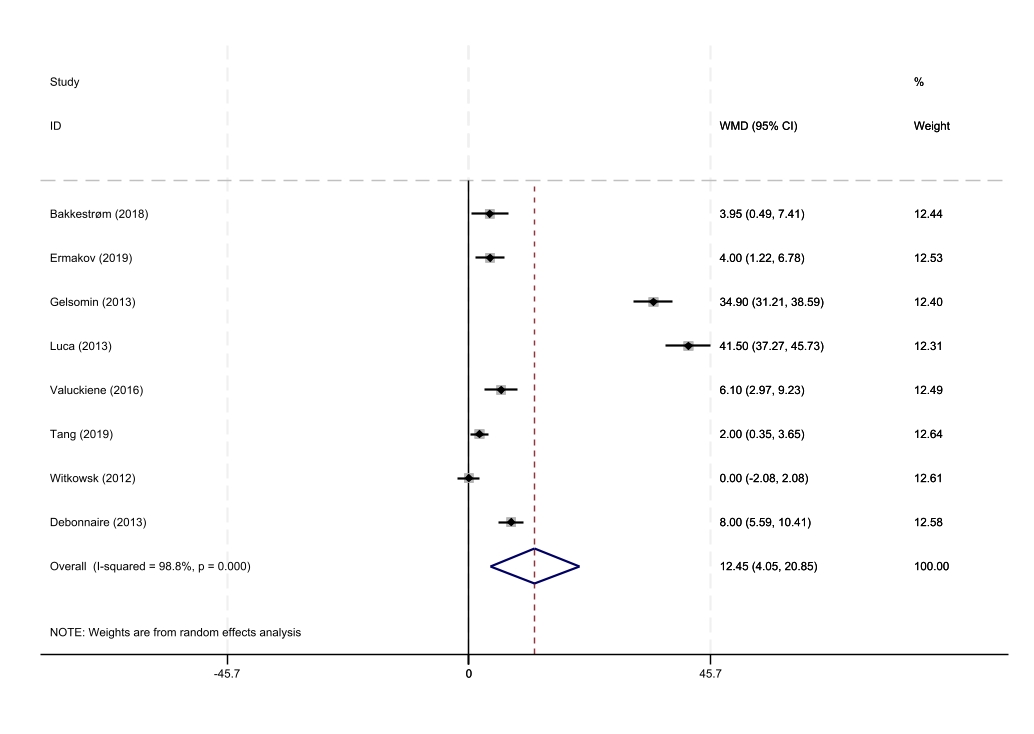

Supplement: Supplementary file 7 [file Image7.jpeg]

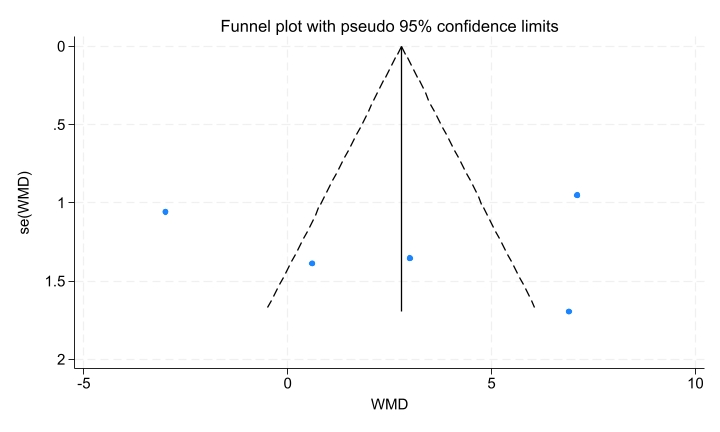

Supplement: Supplementary file 8 [file Image8.jpeg]

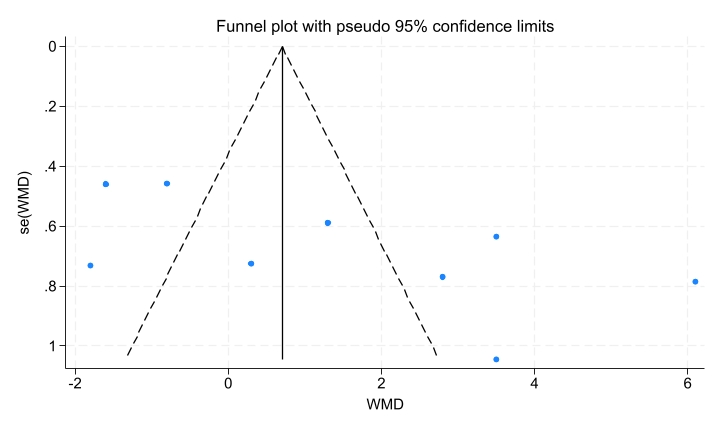

Supplement: Supplementary file 9 [file Image9.jpeg]

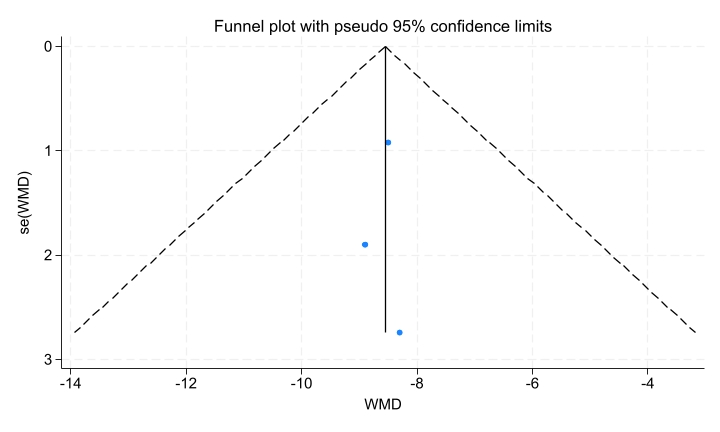

Supplement: Supplementary file 10 [file Image10.jpeg]

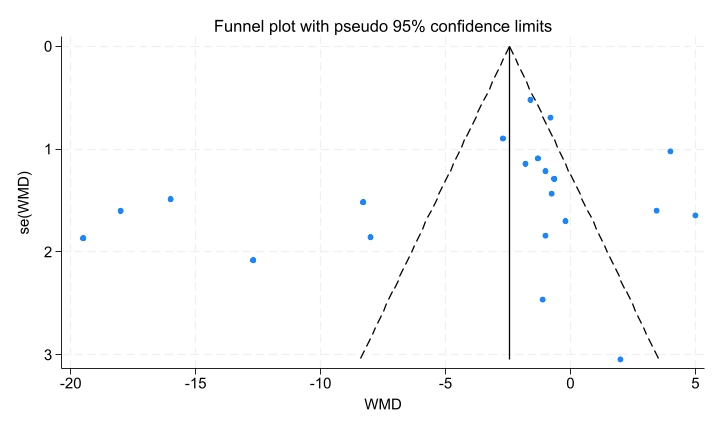

Supplement: Supplementary file 11 [file Image11.jpeg]
